# MRI‐driven Analysis of EEG Connectivity in Alzheimer’s Disease and Behavioral Frontotemporal Dementia

**DOI:** 10.1002/alz.086813

**Published:** 2025-01-09

**Authors:** Giordano Cecchetti, Silvia Basaia, Elisa Canu, Davide G. Curti, Marco Cursi, Edoardo G. Spinelli, Francesca Caso, Giovanna F. Fanelli, Anna Bellini, Giuseppe Magnani, Federica Agosta, Massimo Filippi

**Affiliations:** ^1^ IRCCS San Raffaele Scientific Institute, Milan Italy; ^2^ Vita‐Salute San Raffaele University, Milan Italy

## Abstract

**Background:**

Previous EEG studies highlighted the potential of analyzing neural oscillatory activity for differentially diagnosing Alzheimer’s disease (AD) and frontotemporal dementia (FTD). We recently studied primary progressive aphasia (PPA) variants associated with AD and FTD, revealing distinctive patterns of alterations in linear lagged connectivity (LLC) values within the default mode network (DMN) and the salience network (SN). This study utilizes the same functional MRI‐driven model for source reconstruction to investigate LLC values across typical amnestic AD and the behavioral variant of FTD (bvFTD).

**Method:**

In this cross‐sectional, single‐center study, we consecutively recruited AD, bvFTD, and healthy subjects from the Neurology Unit, IRCCS San Raffaele Scientific Institute, Milan, Italy. Resting‐state 19‐channel EEG data were acquired, and LLC values estimated across 6239 voxels at the whole‐brain level using standardized low‐resolution brain electromagnetic tomography (sLORETA). Subsequently, LLC values were averaged within SN and DMN maps derived from functional MRI data of healthy controls using seed‐based analysis. Network‐based statistics (5000 permutations‐based adjustment) were employed to compare LLC values among study groups.

**Result:**

39 bvFTD patients, 39 AD subjects, and 21 healthy controls were enrolled. AD patients exhibited elevated connectivity at delta frequency band in both DMN and SN (p<0.01), and decreased values at alpha2 band in both DMN (p = 0.04) and SN (p = 0.03) compared to controls. Furthermore, AD patients displayed higher LLC values at theta band than both bvFTD and healthy subjects within both DMN and SN (p = 0.01). BvFTD patients exhibited a significant increase in EEG connectivity at delta band compared to healthy individuals, specifically within SN (p = 0.05). No other significant differences were observed.

**Conclusion:**

LLC findings in AD patients align with our previous studies, indicating a comprehensive disruption of neural oscillatory activity, with theta frequency band emerging as a potential biomarker of AD. Consistent with our study in PPA patients, FTD patients exhibited a connectivity alterations at delta band limited to anterior cerebral regions. EEG holds promise in the differential diagnosis between AD and bvFTD, suggesting a link with underlying pathology.

**Funding**: Foundation Research on Alzheimer Disease. Next Generation EU/National Recovery and Resilience Plan, Investment PE8‐Project Age‐It.

